# Dietary-Derived Exosome-like Nanoparticles as Bacterial Modulators: Beyond MicroRNAs

**DOI:** 10.3390/nu15051265

**Published:** 2023-03-03

**Authors:** Mari Cruz Manzaneque-López, Christian M. Sánchez-López, Pedro Pérez-Bermúdez, Carla Soler, Antonio Marcilla

**Affiliations:** 1 Food & Health Lab, Institut de Ciències dels Materials, Universitat de València, Paterna, 46980 Valencia, Spain; 2 Joint Research Unit on Endocrinology, Nutrition and Clinical Dietetics UV-IIS La Fe, 46012 Valencia, Spain; 3 Àrea de Parasitologia, Departament de Farmàcia i Tecnologia Farmacèutica i Parasitologia, Universitat de València, Burjassot, 46100 Valencia, Spain; 4 Departament de Biologia Vegetal, Facultat de Farmàcia, Universitat de València, Burjassot, 46100 Valencia, Spain

**Keywords:** DELNs, microbiota, miRNA

## Abstract

There is increasing evidence that food is an important factor that influences the composition of the gut microbiota. Usually, all the attention has been focused on nutrients such as lipids, proteins, vitamins, or polyphenols. However, a pivotal role in these processes has been linked to dietary-derived exosome-like nanoparticles (DELNs). While food macro- and micronutrient composition are largely well established, there is considerable interest in these DELNs and their cargoes. In this sense, traditionally, all the attention was focused on the proteins or miRNAs contained in these vesicles. However, it has been shown that DELNs would also carry other bioactive molecules with a key role in regulating biochemical pathways and/or interactions with the host’s gut microbiome affecting intracellular communication. Due to the scarce literature, it is necessary to compile the current knowledge about the antimicrobial capacity of DELNs and its possible molecular mechanisms that will serve as a starting point. For this reason, in this review, we highlight the impact of DENLs on different bacteria species modulating the host gut microbiota or antibacterial properties. It could be concluded that DELNs, isolated from both plant and animal foods, exert gut microbiota modulation. However, the presence of miRNA in the vesicle cargoes is not the only one responsible for this effect. Lipids present in the DELNs membrane or small molecules packed in may also be responsible for apoptosis signaling, inhibition, or growth promoters.

## 1. Introduction

Bacteria are everywhere, including our bodies and foods. Most of these bacteria are harmless or helpful, as the species of bacteria that colonize our digestive system, playing a fundamental role in our health by providing defense against pathogens, aiding in nutrient processing, lowering serum cholesterol levels, and improving the immune functions, among others interactive roles [1,2]. In fact, the modulation of gut microbiota to a more favorable profile has been related to a reduced risk of developing a wide range of metabolic, immunological and neurological disorders [3].

In this sense, one of the main environmental drivers of microbiota composition and function is diet. It has become clear that compounds derived from food may promote gut health, either directly or by modulating the composition and function of the gut microbiota and interacting with factors and/or signaling pathways associated with intestinal immune function [4]. However, knowledge of the impact of specific foods or nutrients upon the intestinal microbiota and its mechanism is still limited. Advances in the knowledge of the interactions between food compounds and specific intestinal bacteria would lead to a better understanding of both positive and negative interactions with dietary habits [5].

In addition, an equilibrate microbiota (eubiosis) is also the first barrier against invasive pathogens or resident opportunists. In fact, an imbalanced gut microbiota (dysbiosis) can facilitate pathogen infection and favor a more virulent evolutionary trajectory for the invading pathogens [6]. In most cases, these pathogens reach the host through food. According to a report by the World Health Organization, it is estimated that, yearly, there is a global outbreak of 600 million foodborne diseases, resulting in 420,000 deaths [7]. Among them, the main cause of foodborne disease is bacteria (66%), and the most common alterations are intoxication and infection [8].

Apart from the repercussions for health, it also has economic repercussions unrelated to health since it is estimated that about 25% of annual food loss occurs from food contamination by foodborne pathogens [9]. For this reason, great efforts from the food industry are being dedicated to the development of strategies to prevent bacteria on food, either by killing or inhibiting microbial growth.

Traditional techniques such as salting, drying, freezing or fermentation are applied to extend the shelf life of food products, but there may be a risk of recontamination. Therefore, there is a continuous need for antimicrobial agents [10]. In this sense, several preservatives are employed by the food industry, mainly synthetic additives. Despite their great efficacy, the preservatives can have undesirable side effects, not only in humans but also by causing changes in the organoleptic and/or nutritional properties of food [11].

For this reason, in the last years, natural antipathogen compounds have been gaining interest in the food industry as food preservatives in order to reduce the use of chemical additives. These natural antimicrobials are produced and isolated from different sources, including plants, animals, and microorganisms [12]. An interesting alternative, put on the table in recent years, is the use of extracellular vesicles (EVs).

All bacteria naturally produce and release these EVs (BEVs) with diverse biological functions [13], and it appears to be a conserved process in both pathogenic and non-pathogenic bacteria [14].

In the 1960s, BEVs were first reported in *Escherichia coli*, but their existence has gained attention recently [15] owing to their important roles such as pathogenesis [16], inter-species, intra-species and inter-kingdom communication [17], stress tolerance [18], and immune stimulation [19].

Regarding this, there are many studies about the important role of BEVs, both produced by gut microbiota [20] or by foodborne bacteria [21], in the progression and severity of bacterial infections due to their interactions with animal host cells [22]. These BEVs are one of the key underlying mechanisms behind the harmful or beneficial effects of many pathogenic, symbiont, or probiotic bacteria [23].

In the same way that bacteria secrete EVs, it has been demonstrated that cells from food of both animal and plant origin also secrete them [24,25], and they could also interact with bacteria, regulating their growth or elimination. In view of this, different studies have speculated that these dietary-derived exosome-like nanoparticles (DENPs) would be the third actor between bacteria and hots, becoming a possible alternative strategy to increase or reduce some specific bacteria. However, the mechanism by which DENPs are involved in transkingdom communication is not clear yet.

Certainly, it is known that this cell-to-cell communication through DENPs is made by the transfer of biologically active molecules [26,27]. Although traditionally, all the attention was focused on the proteins or mRNAs contained [28], in recent years, it has been shown that they would also carry other bioactive molecules with a key role in intracellular communication, such as lipids and small-molecule metabolites [29,30].

In this review, we highlight the impact of DENPs on different bacteria species being able to modulate the host gut microbiome or to reduce the pathogenic bacteria population. We then provide an overview of the mechanism involved, recent progress, future potential, and also the remaining challenges of DENs for different biomedical applications.

## 2. Can DENPs Impact Bacteria? A Glance of Scientific Evidence

The International Scientific Association for Probiotics and Prebiotics (ISAPP) defined prebiotics as a ‘substrate that is selectively utilized by host microorganisms conferring a health benefit’. Substrates considered a prebiotic are, for example, conjugated linoleic acid (CLA), polyunsaturated fatty acid (PUFA), non-digestible oligosaccharides (FOS, GOS), human milk oligosaccharides (HMOS), and phytochemicals [31,32].

These prebiotics are then fermented by microorganisms, and the microbial metabolic compounds produced might be responsible for their health benefits, enhancing the number of commensal bacteria and decreasing pathogenic bacteria [33]. As an example, it has been demonstrated that oligofructose (a dietary fiber type) supplementation in human studies induced specific microbial modifications that were associated with an increase in *Bifidobacterium* abundances [34]. It is well established that bifidobacteria confer positive health benefits to their host via their metabolic activities [35].

DENPs are EVs isolated from fruits or vegetables or from food from animal sources such as milk or honey. They have displayed promising results in different pathologies such as cancer, inflammation, nervous diseases, and musculoskeletal disorders. DENPs, especially from edible plants, are resistant to hard gastrointestinal conditions, thus reaching distant organs and allowing, for example, their interactions with gut microbiota [36,37].

### 2.1. Exosomes-like Nanoparticles from Edible Plants

#### 2.1.1. Ginger Exosome-like Nanoparticles

Ginger (*Zingiber officinale*) is a plant whose rhizome has been widely used as a spice and as medicine. Around 400 bioactive compounds have been discovered in ginger that have shown potential health benefits such as anti-inflammatory, anti-tumor, anti-obesity, and antidiabetic effects, among others [38].

Ginger has been reported to show antimicrobial potential due to gingerol and paradol, shogaols and zingerone, which can inhibit the growth of bacteria and fungi proliferation [39]. It shows a potential activity against many Gram-negative bacteria, such as *Escherichia coli*, *Helicobacter pylori, Pseudomonas aeruginosa, Salmonella Newport*, and Gram-positive bacteria, such as *Staphylococcus aureus, Staphylococcus epidermidis, Bacillus subtilis* or *Bacillus nutto* [40].

On the other hand, ginger can also modulate the microbiota population since 6-gingerol has demonstrated, during in-vitro assay, to be able to promote the adhesion of some probiotics (*Lactobacillus acidophilus* and *Bifidobacterium*) to colonic epithelial cells (NCM460 cells and Caco-2 cells), and continuously exerted probiotics activity [41].

Mu J et al. [42] isolated and purified for the first time ginger exosome-like nanoparticles (GELNs) in 2014. After that, various studies have shown the biological properties of GELNs [27,43,44,45,46,47]. However, only two studies have been focused on the antibacterial properties and microbiota modulation of ginger [48,49]. Table 1 summarizes the actions of GELNs on bacteria.

Teng et al. [48] analyzed tissue distribution of different plant-derived exosome-like nanoparticles (ELNs), detecting GELNs in the gut and feces over a 6-hr period. This fact demonstrated that these ELNs were more likely to stay in the intestine, while grapefruit ELNs preferentially migrated to the liver. Then, GELNs were administered to C57BL/6 mice for a week, and the microbial composition via the 16S rRNA gene (v1-v3 regions) was analyzed. The authors found that GELNs: (a) produced an increase in Lactobacillaceae and Bacteroidales S24-7, (b) had no effect on *B. fragilis* or *E. coli* growth, and (c) inhibited Ruminococcaceae growth and a decrease in Clostridiaceae (Table 1). It was hypothesized that the content and type of phosphatidic acid (PA) lipids of GELN may serve as a signal for preferential uptake by *Lactobacillus rhamnosus* GG (LGG).

This significant role of PA lipids was corroborated by Sundaram et al. [49], who concluded that PA present in the membrane of GELNs interacted with hemin-binding protein 35 (HBP35) on the surface of *Porphyromonas gingivalis* (*P. gingivalis*). In fact, the authors corroborated that the degree of unsaturation of PA plays a critical role in GELN-mediated interaction with HBP35. Consequently, GELNs were taken up by the pathogen leading to inhibition growth.

In the case of LGG, GELN-RNAs interact with a panel of bacteria genes, altering the composition of the gut microbiota. miR167a binds the LGG pilus protein SpaC and downregulates their expression [48]. Regarding aly-miR-159a, gma-miR-166u, and gma-miR-166p inhibited the expression of genes encoding virulence proteins of *P. gingivalis* [49].

Besides miRNA GELNs cargo, other studies have detected shogaol and gingerols in GELNs [27,42,46]. These compounds are volatile phenolic components with potential antimicrobial activities, among others; for example, it has been shown that gingerols analogs displayed inhibitory activity against *P. gingivalis* [50].

These results show that membrane lipids of GELNs could be bacteria-specific, and this lipid-bacteria interaction allows the uptake of these EVs. When binding occurs, the release of the EVs cargo triggers bacterial growth alteration.

#### 2.1.2. Lemon Exosome-like Nanoparticles

Lemon is an edible fruit from the tree *Citrus limon* (L.) Burm. f. Its chemical composition includes phenolic acids, coumarins, carboxylic acids, amino acids, and vitamins (especially vitamin C), to which is attributed their beneficial properties (anticancer, anti-oxidant, anti-inflammatory, antimicrobial, anti-obesity, and antidiabetic) [51].

Lemon exosome-like nanoparticles (LELNs) have been demonstrated to inhibit cancer cell proliferation-inducing apoptotic cell death [52,53] or inhibit the growth of p53-inactivated colorectal cancer cells [54].

Although lemon extracts have shown inhibitory activity against Gram-positive and Gram-negative bacteria [55,56,57,58], no studies have been carried out demonstrating antimicrobial activity from LELNs.

However, it has been reported the use of LELNs as prebiotics. Thus, the modulation capacity by LELNs of probiotics such as *Lactobacillus* strains and *Streptococcus thermophiles* (STH) has been established; LELNs increased the bile resistance of LGG and inhibited *Clostridioides difficile* infection, which is responsible for antibiotic-associated colitis [59].

**Figure 1:**
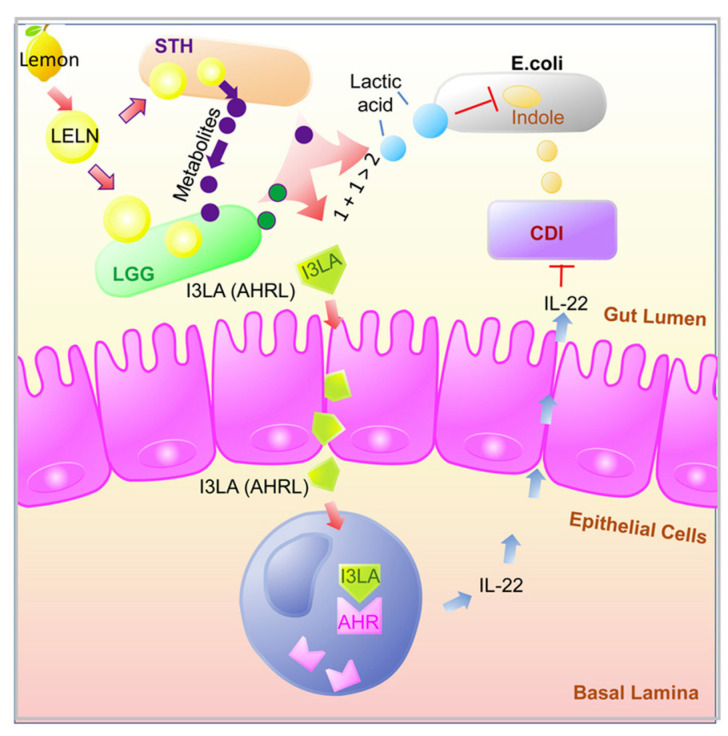
Mechanism of protection of LENLs-treated LGG and STH against *Clostridioides difficile* infection. (Reprinted with permission from Lei C et al. [59] Copyright 2023 American Chemical Society).

As it is shown in Figure 1, this probiotic mix (LGG and STH) previously treated with LELNs increases on the one hand, the AhR ligands indole-3-lactic acid (I3LA) and indole-3-carboxaldehyde (I3Ald), leading to induction of IL-22, and on the other hand, lactic acid, which leads an inhibition of *Clostridioides difficile*. Moreover, metabolites from STH can inhibit the LGG gluconeogenesis pathway to increase the production of I3LA and lactic acid when co-culturing these two strains, thus exhibiting a synergistic effect in protecting against Clostridioides difficile infection [59].

Further, the molecular mechanisms underlying the cross-talk between LELNs and LGG were studied. The authors explored the active component of LELNs that contributes to LGG bile resistance, suggesting that pectin content in LELNs was a major contributor to the increase of this resistance. In fact, previously, it was displayed that pectin from lemon peel improved the viability of LGG in gastric solution by interactions of its pectins with the polysaccharides and proteins on the bacterial cell surface [60].

Raimondo et al. [61] analyzed the LELNs cargo exploring its anti-inflammatory properties. A variety of the flavonoids identified in this study were previously described as possible gut microbiota modulators [62], mainly hesperidin and naringin.

#### 2.1.3. Coconut Exosome-like Nanoparticles

Coconut water is the aqueous part of the coconut endosperm, which is consumed as a beverage. Its nutritional composition contains sugars, sugar alcohols, lipids, amino acids, nitrogenous compounds, minerals, vitamins, organic acids, enzymes, volatile aromatic compounds, and other biochemical compounds [63].

Several reports have shown coconut water as a valuable tool in the treatment of digestive disorders such as treatment of childhood diarrhea, gastroenteritis and cholera [64,65]. This effect has been attributed mainly to three peptides with antimicrobial activities identified in green coconut water (Cn-AMP1, Cn-AMP2 and Cn-AMP3) [66], although the mechanisms of action are unresolved.

Zhao Z et al. [67] isolated coconut exosome-like nanoparticles (CELNs) for the first time. They demonstrated the presence of extracellular miRNAs in coconut water, being the levels higher in mature coconut water than in immature coconut water. These miRNAs were likely to target the human genome and had great potential to affect gene expression (mainly targeting genes associated with metabolic growth).

Additionally, Yu et al. [68] analyzed more precisely the protein and microRNA content of CELNs and investigated the relationship between CELNs and bacteria in order to elucidate the factor affecting bacterial growth. Authors co-incubated *Escherichia coli* K-12 MG1655 and *Lactobacillus plantarum* WCFS1 with CELNs for 4 h and then examined genes (yegH, ptsG, rpoC, and ccpA, which may influence bacterial growth) expression in bacteria. CELNs repressed rpoC and yegH expression levels in MG1655 while elevating those of ccpA after stimulation with CELNs, speculating that the effects of exogenous EVs on bacterial gene expression levels may be attributable to their cargo miRNAs.

Moreover, the authors demonstrated that CELNs could be taken up by bacteria guaranteeing the survival and metabolism of the probiotic bacterium WCFS1.

The findings showed that CELNs could increase the growth of *Escherichia coli* K-12 MG1655 and accelerate the growth of *Lactobacillus plantarum* WCFS1 [67]. WCFS1 is recognized as a probiotic strain due to its immunomodulatory effects [69].

#### 2.1.4. Tartary Buckwheat-Derived Nanovesicles

Tartary buckwheat (Fagopyrum tataricum Gaertn.) is bitter buckwheat that originated in China. It is gaining popularity due to having higher concentrations of certain bioactive phytochemicals, which have protective effects against chronic diseases [70,71]. More concretely, it shows potential gastrointestinal benefits due to its antioxidant and anti-inflammatory capacity [72].

**Figure 2:**
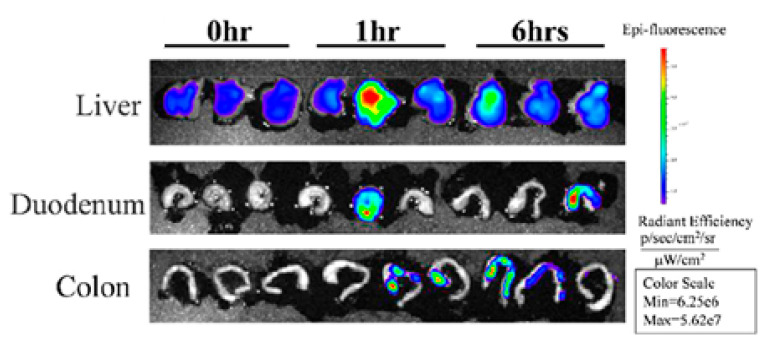
The distribution and absorption of DIR-TBDNs in the liver, duodenum, and colon. (Reprinted with permission from Liu Y et al. [73] Copyright 2023 American Chemical Society).

Liu Y et al. [73] isolated Tartary Buckwheat-Derived Nanovesicles (TBDNs) and evaluated their effects on gut microbiota. In the first step, the authors evaluated the distribution of TBDNs after 1 and 6 h after intragastric administration in mice. As shown in Figure 2, TBDNs were detected in the liver and colon, demonstrating that TBDNs could be absorbed and retained in the intestine, an important prerequisite for TBDNs’ action on the gut microbiota.

They concluded that some microRNAs transported in TBDNs could target functional genes of *E. coli* and *L. rhamnosus,* promoting their growth. Additionally, TBDNs changed the human fecal microecological diversity in comparison with the control group.

It is important to remark that proteins [74,75], flavonoids [76], soluble dietary fiber [77] and resistant starch [78,79] present in Tartary buckwheat could also be filled in TBDNs and involved in this gut microbiota modulation although it has not been studied so far.

### 2.2. Exosomes-like Nanoparticles from Animal-Derived Food

#### 2.2.1. Milk Exosomes

The resistance of human and animal milk-derived EVs (MDEVs) to gastric digestion provides EV entry intact to the human intestine [80], delivering their cargo to intestinal cells and reaching systemic circulation to exert biological activities [81,82].

Due to the MDEVs’ content (proteins, peptides, lipids, coding and non-coding RNAs, oligosaccharides), MDEVs isolated from different species have shown multiple biological effects [81]. The studies researched the effects of MDEVs in the immune response (mainly anti-inflammatory properties), in diseases such as cancer and in other aspects of cell biology [80].

Owing to the critical roles of human breast milk (HBM) in supporting early human growth and development, it has been well-studied over the past decades and continues to attract intense research attention [83]. A particular focus is being paid to the effect that HBM-derived EVs have on the gastrointestinal (GI) tract. Recently, it has been concluded that HBM-derived EVs can alter the intestinal immune response and the subsequent establishment of the microbiota with a cargo capable of influencing the local immune response to bacterial challenge [84,85].

Cow and bovine MDEVs have also shown an impact on the gut microbiota of mice. All studies are summarized in Table 2.

Zhou et al. [86] tested if bovine MDEVs could alter bacterial communities in the murine cecum. A total of 19 families were identified by 16S rRNA sequencing, with an increase of Lachnospiraceae, Firmicutes, and Tenericutes and of Verrucomicrobiaceae in treated mice. The modulation was associated with sex and age. These bacterial families have a direct impact on pathological and physiological conditions. However, the functional consequences of these changes in bacterial communities in the gut and the mechanisms by which these changes contribute to phenotypes of health and disease are unknown.

In another study, the colonic contents from C57BL/6 mice fed with MDEVs for eight weeks were collected, and the microbial composition was analyzed (87), with a finding of an increase in the abundance of *Clostridiaceae, Ruminococcaceae* and *Lachnospiraceae* in MDEVs-treated mice. Moreover, the results showed that *Ruminococcaceae* and *Dehalobacteriaceae* had significant positive correlations with acetate and butyrate, and *Verrucomicrobiaceae* had significant positive correlations with isovaleric and n-valeric. Consequently, MDEVs not only alter the gut microbiota composition but also modulate their metabolites.

In view of the good results, these authors [88] explored the therapeutic effects of MDEVs on mice with induced ulcerative colitis (UC). Firstly, they tested the biodistribution of MDEVs, checking that MDEVs reached the small intestines at 1 h and the colon at 6 h. After 12 h, MDEVs were mainly located in the colon. These findings demonstrated that MDEVs via oral administration could reach the colon and stay for a long time in the gut. Moreover, the results showed that the disturbed gut microbiota in UC was also partially recovered upon treatment with MDEVs.

It is known that gut microbial diversity decreased in DSS-induced colitis. Interestingly, mice treated with MDEVs recovered the relative abundance of bacteria nearly to the level in the control mice and a promising probiotic, *Akkermansia*, was significantly increased.

Du et al. [89] found in female mice, the treatment with MDEVs supposed an enrichment of beneficial microbes such as *Muribaculum* and *Turicibacter* and a decrease of the harmful genera *Desulfovibrio* and *Marvinbryantia* compared with the PBS group. In male mice, MDEVs prominently increased the abundance of *Akkermansia* and decreased the level of *Desulfovibrio*. Although the relative abundance of the identified bacteria was different between female and male mice in response to bovine MDEVs, the overall trend influencing the increase or decrease in the gut microbiota was the same regardless of gender. In this study, the effect of MDEVs on gut microbial metabolites was also determined; in female mice, the proportions of acetic acid, propionate and butyrate were higher in the MDEVs groups, while in male mice, MDEVs significantly increased the proportion of acetic acid, decreased the proportion of propionate, and had no significant role in the butyrate concentration.

It is important to highlight that in these studies, the molecular mechanism explanation of how these MDEVs interact with gut microbiota is missing.

#### 2.2.2. Honey Exosome-like Nanoparticles

Bee-derived products (honey, royal jelly, venom, propolis and pollen) have been reported to exert antimicrobial effects increasing interest in the compounds responsible for these properties [90].

The antibacterial activities of honey are due to lower water activity, high content sugar, glycogenic acid and hydrogen peroxide, both generated by glucose oxidase, peptide defensin-1 (Def-1), and polyphenol compounds [91]. On the other hand, the antibacterial activity of Royal Jelly is a consequence of the synergy between MRJPs, jelleines, royalisin, and trans-10-hydroxy-2-decenoic acid (10-had) fatty acid. At the same time, the antibacterial mechanism of pollen could be exerted by glucose oxidase, phenolic content, phenol content and bioactive compound as fatty acids [90].

Honey-derived EVs (HDEVs) have also been proposed as possibly responsible for these beneficial effects. In fact, previous studies have demonstrated the existence of EVs isolated from pollen, royal jelly and honey and related to antimicrobial effects.

Based on the research of Schuh et al. [92], EVs from Apis mellifera bee pollen, honey (HDEVs) and royal jelly had bacteriostatic, bactericidal and biofilm-inhibiting effects on Staphylococcus aureus. However, the underlying mechanism is still not understood. Assessing the role of EVs within the antibacterial properties of the crude products, the authors found that EVs-depleted bee pollen and royal jelly displayed inhibitory and bactericidal effects at 5% (*v*/*v*), while EVs-rich bee pollen and royal jelly inhibited bacterial growth at 1%. No difference was found for EVs-depleted honey (both 1%). Interestingly, in contrast to its isolated EVs, crude royal jelly displayed a significantly lower biofilm-inhibitory capacity compared with bee pollen and honey.

HDVs’ antibacterial effect has also been tested against the relevant caries-associated Streptococcus mutans and the effect on the commensal Streptococcus sanguinis, which has been described as an important antagonistic species to *S. mutans*. Its prevalence within the biofilm is mostly associated with health conditions [93]. Antibacterial molecules such as MRJP1, defensin-1 and jellein-3 were found as intravesicular cargo. Similarly, Chen et al. [94] identified MRJP1, 2, 4, 8, and 9, but not Jellein-3 or def-1 as HDEVs cargo.

After bacteria incubation with different HDEVs concentrations [93], results demonstrated that, despite leading to an antibacterial effect on both strains of oral streptococci, HDEVs had a pronounced activity against *S. mutans* compared to *S. sanguinis*. It could be associated with mechanical alterations resulting in membrane damage.

Bactericidal effect on *S. aureus* of royal jelly-derived EVs (RJDEVs) had been confirmed in vivo [95]. After 48 h, in mice treated with collagen gel with RJDEVS, no bacteria were detected. In this study, MRJP1, def-1 and Jellein-3 were identified in the EVs.

These findings suggest that MRJP1, def-1 and Jellein-3 are responsible for the antibacterial function of HDEVs. However, in the absence of a metabolomics analysis of the cargo, it is not possible to say that peptides alone are responsible for the observed effects.

The bactericidal effect on *S. aureus* of royal jelly-derived EVs (RJDEVs) has been confirmed in vivo [95]. After 48 h, in mice treated with collagen gel with RJDEVS, no bacteria were detected. In this study, MRJP1, def-1 and Jellein-3 were identified in the EVs.

These findings suggest that MRJP1, def-1 and Jellein-3 are responsible for the antibacterial function of HDEVs. However, in the absence of a metabolomics analysis of the cargo, it is not possible to say that peptides alone are responsible for the observed effects.

## 3. Conclusions

This review has summarized recent research on the functionality of DELNs as a bacterial modulator, and we can conclude that the evidence strongly conveys that DELNs have a direct impact on bacteria growth.

While it is true that most studies are focused on miRNA-EVs as the key molecules responsible for the interaction between DELNs and bacteria, other bioactive molecules packed in EVs, i.e., lipids integrating the membrane, are gradually arising as alternative key compounds involved in gut microbiota modulation.

Numerous studies have highlighted the role of microbiota in health and the importance of maintaining eubiosis. For this reason, there is a crucial need for a better understanding of how DELNs interact with bacteria and how to induce a microbiota modulation useful for disease treatment. For this, further investigation is necessary prior to deliver EVs-based therapies clinically.

## Figures and Tables

**Table 1:** GELNs susceptibility of bacteria.

Reference		Direct			Indirect *	
		Inhibit	Promoted	No Effect	Inhibit	Promoted
[48]	*Lactobacillus rhamnosus (LGG)*		x		-	-
	*Lactobacillus reuteri (L. reuteri)*		x		-	-
	*Lactobacillus murinus (L. murinus)*		x		-	-
	*Bacillus fragilis (B. fragilis)*			x	x	
	*Escherichia coli (E. coli)*			x	x	
	*Ruminococcaceae sp. (TSD-27)*	x			-	-
	*Listeria monocytogenes (L. monocytogenes)*	-	-	-	x	
[49]	*Porphyromonas gingivalis (P. gingivalis)*	x			-	-
	*Fusobacterium nucleatum (F. nucleatum)*	x			-	-
	*Prevotella intermedia (P. intermedia)*	x			-	-
	*Aggregatibacter actinomycetemcomitans (A. actinomycetemcomitans)*	x			-	-
	*Streptococcus gordonii (S. gordonii)*			x	-	-

* Metabolic products from GELNs-treated LGG; (x) Effect; (-) No effect.

**Table 2:** MDEVs have shown an impact on the gut microbiota of mice.

Ref.	Sample	Isolation	Animal	EVs	Conclusions
[86]	Bovine Milk	Differential ultracentrifugation	Female and male C57BL/6 3 weeks	Exosome and RNA-depleted (ERD) VS exosome and RNA-sufficient (ERS) diets	Sex alone does not affect microbial communities. 7 weeks. Two unclassified families from phylum *Firmicutes* were more abundant in ERD mice than in ERS mice. 15 weeks. one of the two unclassified families from the phylum of Firmicutes and another unclassified family from the phylum Tenericutes were more abundant in ERS mice compared with ERD mice 47 weeks. the family of Verrucomicrobiaceae was more abundant in ERD mice compared with ERS mice, whereas Lachnospiraceae and two unclassified families from phyla Firmicutes and Tenericutes were more abundant in ERSmice than in ERD mice
[87]	Raw milk	Chymosin or hydrochloric acid treatment combined with ultracentrifugation/ultrafiltration	Specific-pathogen-free female C57BL/6 3 weeks	Low mEVs VS Middle mEVs VS High mEVs VS control PBS	Increase of Clostridiaceae, Ruminococcaceae Lachnospiraceae with EVs (with an increase of mEVs) and decrease in S24_7.
[88]	Raw milk from Holstein cows	Chymosin combined with ultracentrifugation	Specific-pathogen-free male C57BL/6 7–8 weeks	Control group VS DSS group VS DSS + mEVs-Low dose	Genus level: Depletion of Enterorhabdus and unclassified_Bacteroidia in the DSS group, but recovered in the mEVs group Family level: Increased Enterococcaceae and Desulfovibrionales-unclassified Desulfovibrionaceae in the DSS group but unchanged in the mEVs group
[89]	Bovine raw milk	Ultracentrifugation	Specific-pathogen-free female and male C57BL/6 6–8 weeks	High EVs VS Middle EVs VS Low EVs VS Control PBS	Female Genus: increase Muribaculum, Turicibacter. Decrease Desulfovibrio, Marvinbryantia. Male Genus: increase Akkermansia. Decrease Desulfovibrio. Phylum: increased Verrucomicrobia and Cyanobacteria

## Data Availability

Not applicable.
